# T Lymphocyte Density and Distribution in Human Colorectal Mucosa, and Inefficiency of Current Cell Isolation Protocols

**DOI:** 10.1371/journal.pone.0122723

**Published:** 2015-04-09

**Authors:** Gloria Cuevas Preza, Otto O. Yang, Julie Elliott, Peter A. Anton, Maria T. Ochoa

**Affiliations:** 1 Department of Dermatology, Keck School of Medicine, University of Southern California, Los Angeles, CA, United States of America; 2 Department of Medicine, David Geffen School of Medicine, University of California Los Angeles, Los Angeles, CA, United States of America; 3 Department of Microbiology, Immunology, and Molecular Genetics, David Geffen School of Medicine, University of California Los Angeles, Los Angeles, CA, United States of America; 4 UCLA AIDS Institute, David Geffen School of Medicine, University of California Los Angeles, Los Angeles, CA, United States of America; 5 AIDS Healthcare Foundation, Los Angeles, CA, United States of America; Harvard Medical School, UNITED STATES

## Abstract

Mucosal tissues are critical immune effector sites containing complex populations of leukocytes in a tissue microenvironment that remains incompletely understood. We identify and quantify in human distal colorectal tissue absolute mucosal CD3^+^ lymphocytes, including CD4^+^ and CD8^+^ subsets, by direct visualization using immunohistochemistry (IHC), immunofluorescence (IF), and an automated counting protocol (r^2^=0.90). Sigmoid and rectal mucosal tissues are both densely packed with T lymphocytes in the mucosal compartment. Both compartments had similar densities of CD3^+^ T lymphocytes with 37,400 ± 2,801 cells/mm^3^ and 33,700 ± 4,324 cell/mm^3^, respectively. Sigmoid mucosa contained 57% CD3^+^CD4^+^ and 40% CD3^+^CD8^+^ T lymphocytes which calculates to 21,300 ± 1,476/mm^3^ and 15,000 ± 275/mm^3^ T lymphocytes, respectively. Rectal mucosa had 57% CD3^+^CD4^+^ and 42% CD3^+^CD8^+^ or 21,577 ± 332, and 17,090 ± 1,206 cells/mm^3^, respectively. By comparison, sigmoid mucosal biopsies subjected to conventional collagenase digestion, mononuclear cell (MMC) isolation and staining for flow cytometry yielded 4,549 ± 381/mm^3^ and 2,708 ± 245/mm^3^ CD4+ and CD8+ T lymphocytes. These data suggest only ~20.7% recovery compared to IHC results for these markers. Further studies will determine if this reflects a selective bias in only CD3^+^, CD4^+^ and CD8^+^ T cells or can be generalized to all flow-analyzed cells from mucosal tissues for phenotyping and functional testing.

## Introduction

The mucosal immune compartment is the largest lymphoid reservoir of the body, a critical compartment in immune-protection such as in the setting of various infectious diseases and cancer. Dysfunction can result in certain autoimmune diseases (inflammatory bowel diseases) or increased susceptibility to infection. Technical advances have made *in situ* imaging and quantification of labeled cell types feasible, enhancing efforts to characterize immune responses in lymphoid organs and peripheral tissues. As direct quantification has been a time-intensive and variable approach, isolation of mucosal mononuclear cells (MMCs) with subsequent flow cytometric quantification has been the *de facto* standard to characterize MMC populations of interest.

While long suspected, it has become clear that isolating cells by the disruption of their microenvironment can profoundly alter their properties [1–2]. Microscopy *in situ* provides critical information including accurate tissue localization and spatial relationships of immune cell populations [3]. Standardizing automated approaches to detect and quantify mucosal-based cells of interest is critically needed. Given improved automated approaches to quantifying cells *in situ*, we sought to investigate the numbers and distributions of mucosal CD3^+^, CD4^+^ and CD8^+^ T lymphocyte populations in healthy human distal colon and rectum. The numbers of these cells in tissue have been debated and under-described in the scientific literature [4–7]. To accomplish this comparative but descriptive study with minimal additional resources, we utilized numbers of mucosal CD3^+^, CD4^+^ and CD8^+^ T lymphocyte populations quantified by flow cytometry from healthy controls in other NIH-funded studies, leveraging that data to compare with counts via IHC using newly recruited subjects through the UCLA CFAR’s Mucosal Immunology Laboratory Core.

## Materials and Methods

### Study participants

Data acquired for comparison within this paper was derived from mucosal biopsy samples acquired from two separate NIH-funded, UCLA-based studies: Effects of Aging on GALT (AG032422; R. Effros, UCLA: PI) (the consent form approves use of samples/data for other mucosal immune-related, IRB-approved studies) and the UCLA CFAR’s Mucosal Immunology Laboratory Core (AI2869). This particular study was approved both by the USC and UCLA Offices of the Human Research Protection Program Institutional Review Board and all subjects provided written informed consent.

### Collection of human sigmoid colon and rectal mucosal tissue

Gastrointestinal mucosal biopsies were freshly-acquired from the sigmoid colon and rectum as previously described [8]. Briefly, healthy HIV-1-seronegative participants without evidence of rectal sexually transmitted diseases (clinically screened for *Chlamydia trachomatis* and *Neisseria gonorrhoeae*) or chronic gastrointestinal disorders were recruited to undergo flexible sigmoidoscopy with biopsies. Using large-cup endoscopic biopsy forceps (Microvasive Radial Jaw #1589, outside diameter 3.3 mm), biopsies (approximately 1mm deep) were obtained at 5–8 cm from the anal verge (“rectal colon”) and then from 25–30cm (“sigmoid colon”). Whether in the past (archived data) or present, biopsy specimens were immediately placed in RPMI 1640 and quickly transported to the laboratory, for either preparation for flow cytometry phenotype characterization (with isolation of mucosal mononuclear cells (MMCs)) or embedded in OCT medium (Ames Co., Elkhart, IN) and then snap-frozen in liquid nitrogen (~30–45 minutes from acquisition to freezing). On average, biopsy sizes were ~8mm x 3mm x 1mm.

### Section preparation and immunohistochemical tissue staining of T lymphocytes

Cryostat sections (4 μm thick) were acetone-fixed, blocked with normal horse serum and then sequentially incubated with the indicated murine anti-human monoclonal antibodies and corresponding isotype controls, biotinylated horse anti-mouse IgG, and an avidin/biotin-peroxidase. Tissues were counterstained with Vector Hematoxylin QS (Vector Labs; Burlingame, CA) for light microscopy (Olympus BX51 microscope, Central Valley, PA, equipped with an Olympus America camera). The following mouse anti-human antibodies were used: anti-CD3 (clone HIT3a; BD Pharmingen, San Diego, CA), anti-CD4 (clone OKT-4; eBioscience, San Diego, CA), anti-CD8 (clone C8/144B; DAKO, Carpinteria, CA), anti-CD45 (clone 2B11+PD7/26/16; AbD Serotec, Raleigh, NC), anti-CD1a (clone NA1/34-HLK; AbDSerotec, Raleigh, NC), and CD68 (clone EBM11; DAKO, Carpinteria, CA).

### Manual *in situ* enumeration of tissue T lymphocytes

Immunostained cells in epithelium and lamina propria including lymphoid aggregates were enumerated as previously described [9]. Slides were scanned using an Aperio ScanScope (Leica Biosystems, Vista, CA); images were analyzed using Leica’s membrane algorithm in Imagescope software. On each slide, three separate, non-overlapping regions of interest were circumscribed for counting. Each distinct area included 5–10 glands with associated lamina propria and any lymphoid aggregates. Labeled cells were then counted manually by visualization using Image Scope imaging software from Leica Technologies, Inc. (Vista, CA) at 40x magnification: the counts were normalized to cells/mm^2^ by taking the cell count and dividing by the area of the region of interest (mm^2^) then averaging the 3–5 separate regions of interest. The concentration of cells per cubic mm (mm^3^) was then calculated by multiplying the cell count/mm^2^ by 250 (this is derived based on there being approximately 250 slices per biopsy when each sectioned slice is made of 4μm sections in 1mm thickness) and dividing by 3.356 (correction factor based on the average number of sections in which a T lymphocyte appears in 4μm thick sections, S1 Fig). For statistical analyses, averages of at least three sections from five biopsies were utilized (Student t-test).

### Automated *in situ* enumeration of T lymphocytes in colorectal mucosa

Using the same digitally analyzed/recorded areas from the same tissue sections already manually counted, we used the IHC membrane algorithm from Leica Technologies, Inc. (Vista, CA) to quantify membrane staining to determine the number of CD3^+^ mucosal cells/mm^3^. For membrane staining, various input parameters were optimized and validated for each tissue sample and isotype controls. The total number of labeled cells were graded as +1, +2, and +3 (data not shown) for intensity based on three separate areas surrounding similar 5–10 glands including lamina propria and lymphoid aggregates (when present). The automated-derived counts were used to calculate cells/mm^3^ (as above for the manually-counted calculations using the same sections/areas). For statistical analysis we performed paired t tests on counts obtained by manual counting and compared them to the counts from the same regions obtained by algorithm analysis. There were no significant differences between counts obtained by manual counting compared to counts obtained by algorithm. Sigmoid p = 0.552 and rectum p = 0.517.

### Two color immunofluorescence of human mucosal tissues

Immunofluorescence was performed by serially incubating cryostat-prepared tissue sections (1–2 sections away from the above sectioned/enumerated counts; same biopsy samples) with mouse anti-human monoclonal antibodies against CD3^+^, CD4^+^, and CD8^+^ as above, followed by incubation with isotype-specific, fluorochrome-labeled, goat anti-mouse antibodies (Alexa 488 and Alexa 568, Molecular Probes, Eugene, OR). Controls included were isotype-matched control mouse antibodies as well as specific mouse antibodies with a mismatched secondary antibody. Slides were mounted with ProLong Gold anti-fade reagent with a nuclear marker DAPI (Molecular Probes; Eugene, OR). Images were obtained using confocal laser microscopy using a Leica-TCS-SP5 MP inverted confocal laser-scanning and two-photon laser microscope (Heidelberg, Germany) fitted with DPSS-diode (561 nm), argon (488 nm), and two-photon laser (Spectra-Physics Millenia X 532 diode pump laser and Tsunami picosecond Ti-sapphire laser) at the Craniofacial Center for Molecular Biology at USC. Sections were illuminated with 488/568 nm for green/red and DAPI for nuclear label. Images of specimens with Alexa 488, Alexa 568 were recorded sequentially through the spectral emission filters: from 500–550nm for Alexa 488; 580–700nm for Alexa 568; 410 to 490nm for DAPI). Pairs of single images were superimposed for co-localization analysis. All micrographs were compiled in serial Z-stack images 0.1–1.0 μm apart and analyzed and normalized using LAS AF Lite software, Leica Microsystems (Heidelberg, Germany). The number of CD3^+^/CD4^+^ and CD3^+^/CD8^+^ double positive cells was assessed in three high-power fields from 3–5 sections of sigmoid and rectal biopsies. The percentage of double positives was calculated for each section in three areas of 450 x 450 μm^2^; the number of double-positive cells/mm^3^ was determined by taking the percentage of double positives from the total absolute number of CD3^+^ cells obtained from manual counting.

### Isolation and flow cytometric analysis of mucosal mononuclear cells (MMC)

MMCs were isolated from 48 seronegative subjects (30 biopsies per individual) using a previously optimized protocol [8,10–13]. Briefly, the 30 freshly acquired sigmoid biopsies were washed once in RPMI 1640 medium and distributed equally to two 50 ml conical centrifuge tubes (15 biopsies per tube). Samples were incubated in 20–25 ml RPMI/7.5% FCS containing 0.5-mg/ml collagenase type II (Sigma-Aldrich, St. Louis, MO) for 30 minutes in a 37°C water bath, with intermittent shaking. Tissue fragments were further disrupted by forcing the suspension 5–6 times through a 10 cc disposable syringe attached to a blunt-ended 16-gauge needle (Stem Cell Technologies, Vancouver, B.C.). The entire suspension was then passed through a 70 μm sterile plastic strainer (Falcon 2350) to remove free cells while concentrating the remaining tissue fragments. Free cells were immediately washed twice in RPMI 1640 medium to remove excess collagenase, prior to being resuspended in 500ul–1000ul RPMI with 10% FBS and set aside on ice. Remaining tissue fragments were returned to a 50 ml conical tube and the entire procedure, including the 30-minute collagenase incubation repeated two additional times or until tissue fragments were no longer intact. Free cells were combined at the completion of the digestion and the resultant single cell suspension used for flow cytometric studies.

Lymphocyte subsets in MMC preparations were quantified by Trucount (BDIS, Mountain View, CA) according to the manufacturer’s instructions. Briefly, 20μl of Multitest (BDIS, Mountain View, CA) reagent containing either CD3-FITC/CD8-PE/CD45-peridinin chlorophyll protein (PerCP)/CD4-allophycocyanin (APC) or CD3-FITC/CD16+CD56-PE/CD45-PerCP/CD19-APC was added to two Trucount tubes (contain a known number of fluorescent beads) followed by 100 μl of MMC single cell suspensions. Following incubation for 15 minutes in the dark, any residual red blood cells were lysed and the remaining cells were simultaneously fixed by the addition of 450 μl of FACS lysing solution (BDIS, Mountain View, CA). Samples were analyzed on a FACS Calibur flow cytometer using CellQuest software. A viable lymphocyte gate was established using CD45 and side scatter parameters. T-cells were further delineated using a CD3 + gating strategy, with the CD4+/CD8+ subsets determined by further gating of the well defined populations, as previously described [10]. The absolute numbers (cells/μl) of CD45^+^CD3^+^CD4^+^, CD45^+^CD3^+^CD8^+^, CD45^+^CD3^-^CD16^+^CD56^+^, and CD45^+^CD3^-^CD19^+^ lymphocytes were then quantified by dividing the number of positive cellular events by the number of bead events and multiplying the result by the bead concentration. ([Antibody positive cells/Total bead events] x Beads/ul). Absolute numbers per biopsy were derived from the pooled sample of 30 biopsies.

## Results

### Similarity to compared study populations

The total study population was comprised of 54 HIV sero-negative, healthy participants. The study participants from the previously conducted flow cytometry study was comprised of 48 subjects (44 male; 4 female) with a median age of 41 (range: 19–66) with the following racial distribution: Caucasian (N = 14), African-American (N = 18), Hispanic (N = 8), unknown (N = 8). The study participants of newly recruited CFAR CORE subjects for IHC comparisons consisted of 5 sigmoid and 6 rectal, one rectal biopsy was not suitable for analysis, so it was excluded, this gave a total of 5 sigmoid and 5 rectal biopsies for analysis. These samples were collected from 6 male subjects with a median age of 25 (range: 19–47) with the racial distribution of: Caucasian (N = 2), 1 African-American (N = 1), Asian (N = 1), unknown (N = 1). There were no discernable differences between these two small study populations.

### Both sigmoid and rectal mucosa are densely populated with CD3^+^ lymphocytes

**Fig 1:**
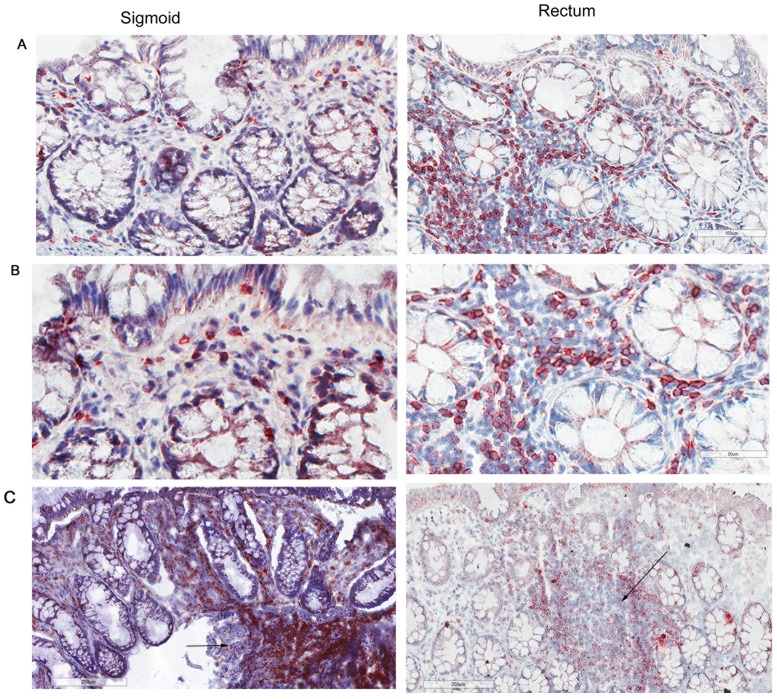
CD3^+^ lymphocytes are similar in normal human sigmoid colonic and rectal mucosa. Sigmoid colonic and rectal mucosal sections immunostained for CD3. The membrane red/brown colors denote positive staining (red/brown, AEC chromogen). Specimens were counterstained with hematoxylin. (A)Upper panel original magnification 200x (B) lower panel 400x (C) lower magnification showing distribution of lymphoid aggregates in both sigmoid and rectum (100x). Arrows represent lymphoid aggregates.

To establish an average size of mucosal biopsies, measurements of 20 biopsies from two persons (10 from each anatomic site) were assessed, yielding a mean volume of 20.2 ± 5.9 mm^3^ (data not shown). By both manual and automated counting methods, CD3^+^ lymphocytes were widely distributed throughout the epithelia, lamina propria and clustered in lymphoid aggregates (Fig 1a–1d). Lymphoid aggregates were identified in ~60% sections from both sigmoid and rectal mucosa samples; there were ~1.33 lymphoid aggregates/section (Fig 1c). The distribution of CD45 was assessed in the same tissues and was found to also be widely distributed similar to CD3^+^ T lymphocytes. CD45 is present on all differentiated hematopoietic cells, except red blood cells and plasma cells, which include T lymphocytes, monocytes, and some dendritic cells (S2 Fig).

**Fig 2:**
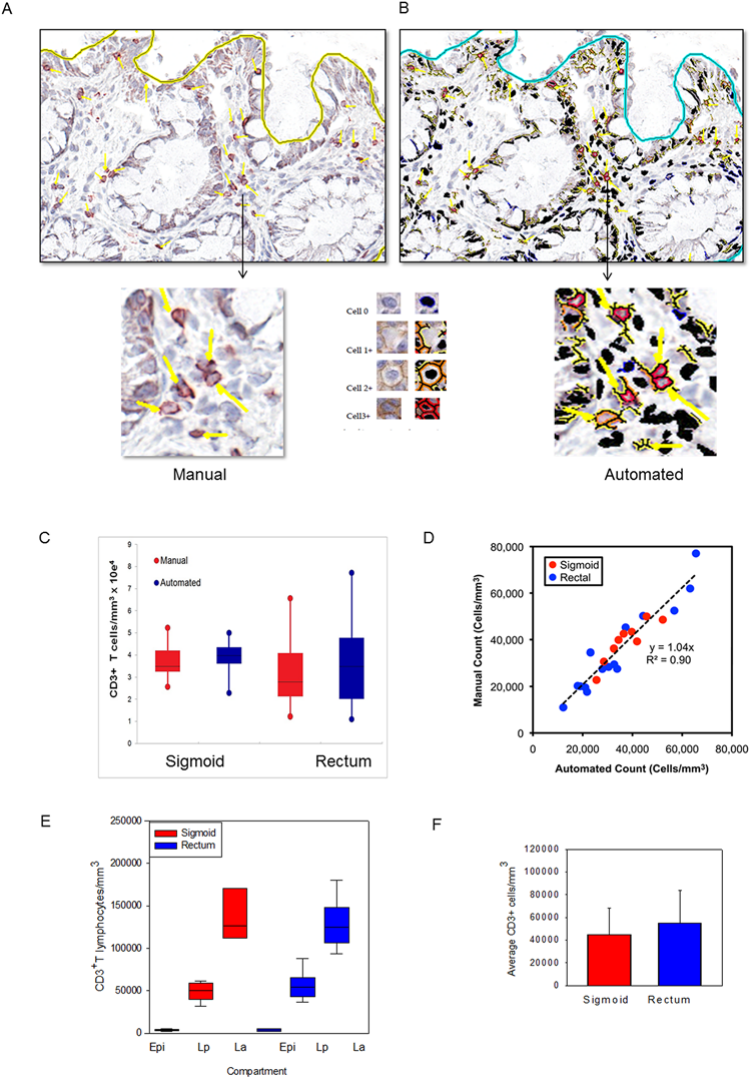
Application of an automated algorithm to quantitate T lymphocytes in immunostained sigmoid colonic and rectal mucosa. A membrane detection algorithm was applied to CD3-immunostained sigmoid colonic and rectal mucosal biopsies from 5 healthy donors (5 sigmoid colon, 5 rectum). (A) Digital images with AEC chromogen staining for CD3 and hematoxylin counterstaining. (B) Mark-up images with red, orange, and yellow pixels depicting immunopositive cells (strong, moderate, and weak intensity, respectively), and black pixels depicting nuclei for immunonegative cells. Bar = 100 μm. Arrowheads denote manual enumeration. (C) Tissue concentrations of T lymphocytes obtained using both methods and analyzed by paired t test. (D) Correlation between counts obtained by manual and automated enumeration of the same regions. R^2^ value = 0.90 (E) Tissue concentrations of T lymphocytes in epidermis (Epi), lamina propria (Lp), and lymphoid aggregates (La; when present) of sigmoid colon and rectum. (F) Average T lymphocyte concentrations obtained from averaging the various compartments from the sigmoid colon and rectum.

Manual counting revealed overall densities of 37,400 ± 2,801/mm^3^ and 33,700 ± 4,324/mm^3^ in sigmoid colonic and rectal mucosa, respectively (Fig 2c). Using an average diameter of 12 μm for an activated T lymphocyte (corresponding to a volume of 905 μm^3^ per cell), these counts suggest that ~3.38 ± 0.25% and ~3.05 ± 0.39% of the sigmoid and rectal mucosal tissue biopsy volumes, respectively, are composed of T lymphocytes. Based on assumptions that mucosal thickness (up to but not including muscular layers) to be 1.5mm with the sigmoid colon ~35cm long with an inner diameter of 3.5cm (total mucosal volume of 350 x [π x 19.0^2^ - π x 17.5^2^] = 6020 mm^3^), and rectum to be 5cm in length [14] with an inner diameter of 5cm (total mucosal volume of 50 x [π x 26.5^2^ - π x 25.0^2^] = 8494 mm^3^), these counts equate to 2.25 ± 0.17 x 10^9^ and 4.09 ± 0.52 x 10^8^ total T lymphocytes in the sigmoid and rectal mucosa respectively, or 2.66 ± 0.21 x 10^9^ total for the entire rectosigmoid mucosa.

### Automated counting protocol yields are similar to those from direct, manual counting

To assess the validity of the membrane Leica algorithm automated counting protocol (Leica Biosystems Technologies, Inc.), the same specimens manually counted above were re-analyzed (Fig 2a–2c). After optimization, this protocol counted CD3^+^ cells with a high degree of correlation to manual counting. Automated versus manual counting were compared for three representative fields each from sigmoid and rectal mucosal biopsies from five persons. The best-fit line by linear regression yielded an overall r^2^ value of 0.90, with a slope of 1.04 and y-intercept of 0 (Fig 2d). These data validated a rapid and highly reliable approach to automated counting of cells after immunoperoxidase staining for these markers. Analysis of the epithelium, lamina propria, and lymphoid aggregates as separate compartments yielded similar averages per mm^3^ of CD3^+^ cells (Fig 2e and 2f).

#### CD4^+^ and CD8^+^ T lymphocytes are similarily distributed in sigmoid and rectal mucosal tissues

**Fig 3:**
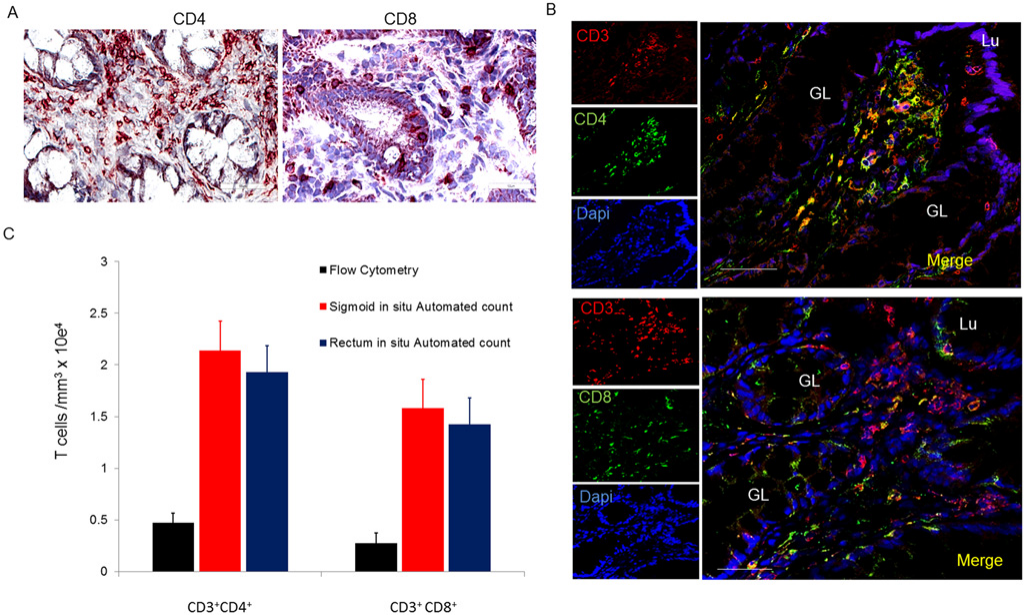
Distribution and number of CD4^+^ and CD8^+^ T lymphocytes in sigmoid mucosal and rectal mucosa. (A) Immunoperoxidase for CD4 and CD8 with hematoxylin counterstaining. (B) Distribution of CD4^+^ and CD8^+^ cells in lymphoid aggregates. (C) Confocal microscopy of CD3^+^CD4^+^ and CD3^+^CD8^+^ subsets of T lymphocytes. Bar = 50μm. (D) Densities of CD4^+^ and CD8^+^ T lymphocytes compared to densities obtained from flow analysis. GL = gland; Lu = lumen

The localization and number of CD4^+^ and CD8^+^ subsets of T lymphocytes were characterized *in situ* using IHC and manual counting. Both CD4^+^ and CD8^+^ T lymphocytes were widely distributed in the lamina propria, (Fig 3a) and, as expected, more so in denser areas of lymphoid aggregates (Fig 3b). CD8^+^ and some CD4^+^ T lymphocytes were additionally located sparsely intraepithelially, in agreement with prior observations [7,15]. To confirm CD4^+^ and CD8^+^ lymphocytes (and not macrophages and dendritic cells which also express CD4^+^ and CD8^+^ receptors), we used immunofluorescence (IF) dual staining with CD3^+^ (Fig 3c) followed by manual counting of double positives CD3^+^CD4^+^ and CD3^+^CD8^+^ cells. In the rectum, CD3^+^CD4^+^ T lymphocytes accounted for 57% of T lymphocytes (absolute numbers: 21,577 ± 332 cells/mm^3^); and 42% were CD3^+^CD8^+^ T lymphocytes (absolute numbers: 17,090 ± 275 cells/mm^3^). By volume, 1.95% (± 0.03%) and 1.55% (± 0.11%) of the rectal mucosa is composed of CD4^+^ and CD8^+^ T lymphocytes, respectively. Similarly, in the sigmoid, CD3^+^CD4^+^ T lymphocytes accounted for 57% CD3^+^CD4^+^ T lymphocytes (absolute number 21,300 ±1,476 cells/mm^3^) and 40% CD3^+^CD8^+^ (15,000 ± 275/mm^3^) T lymphocytes. This corresponds to 2.07 ± 0.13% and 1.37 ± 0.02% of the sigmoid colonic mucosa being composed of CD4^+^ and CD8^+^ T lymphocytes respectively. Based on the assumed volumes for sigmoid and rectal mucosae above, these figures suggested that they contain 1.38 ± 0.09 x 10^9^ and 2.62 ± 0.04 x 10^8^ CD4^+^ T lymphocytes, and 9.10 ± 0.17 x 10^8^ and 5.41 ± 0.38 x 10^8^ CD8^+^ T lymphocytes respectively. Efforts to adjust numbers of T lymphocytes subsets to biopsy volume, enables the reporting of the “# of cells/biopsy”. Consistent with our recent report, there was a significant population of CD4^+^ but CD3^-^ cells that co-expressed CD68 and CD1a indicating they were of monocyte/macrophage lineage (data not shown) [9].

#### Flow cytometric counting of MMC markedly underestimates densities of CD4^+^ and CD8^+^ T lymphocytes

**Fig 4:**
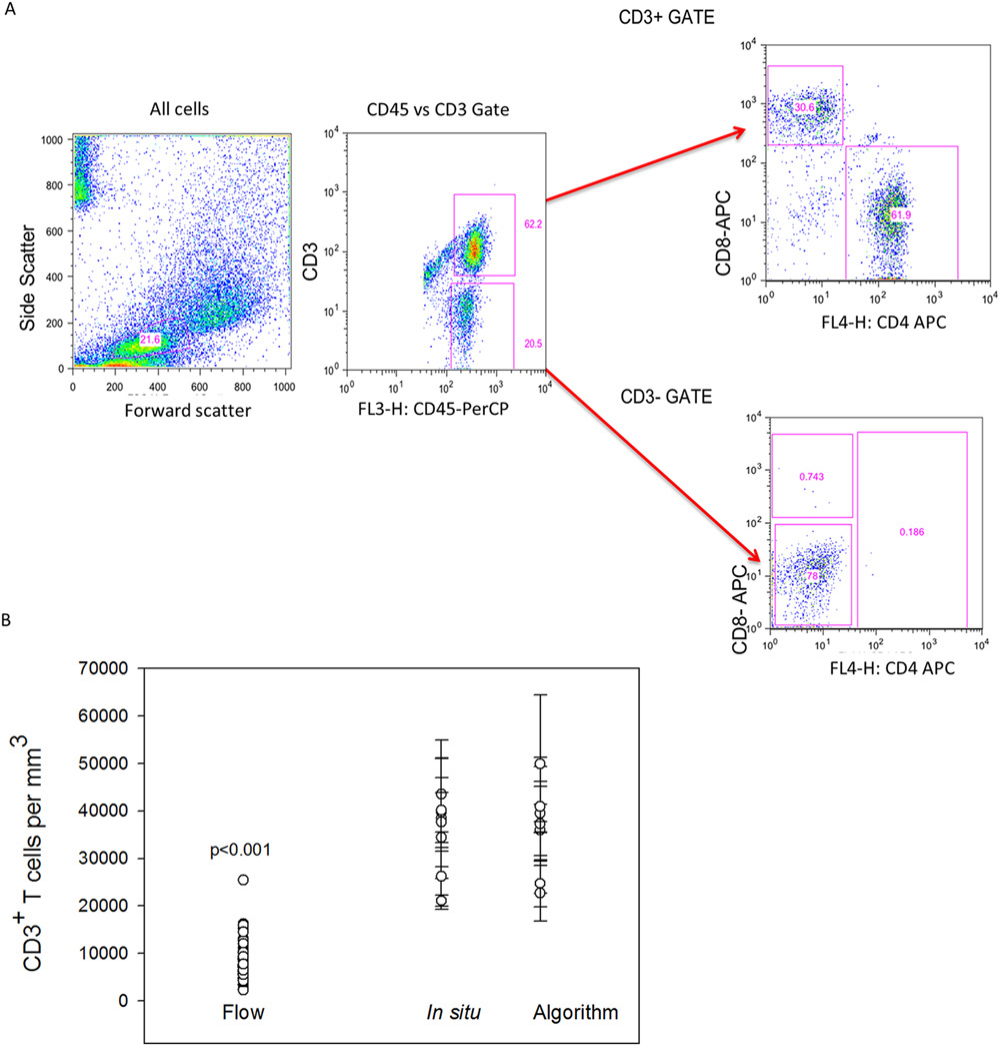
Comparison of automated counts of *in situ* sigmoid colonic and rectal mucosal T lymphocytes versus isolation and flow cytometric counting. Mucosal CD3^+^ T lymphocytes were isolated by mechanical tissue disruption and collagenase digestion. (A) Gating strategy for flow cytometric analysis of isolated lymphocytes. (B) Comparison of the mean numbers of sigmoid colonic mucosal CD3^+^ lymphocytes isolated from 30 sigmoid biopsies from similar persons versus automated (n = 48) and manual counting of 5 sigmoid biopsies and 5 rectal biopsies from 5 subjects. Dots represent the means of 3–5 regions analyzed with bars representing standard error of the means. (Mann-Whitney Rank Sum test p<0.001).

Only sigmoid biopsies were used to compare differences in counted yields “per biopsy” between IHC and flow cytometry with TruCount beads. Biopsy samples were processed using standard methodology (with collagenase II digestion) to isolate MMC from sigmoid colonic mucosa of healthy individuals. The flow cytometry yield of CD4^+^ and CD8^+^ T lymphocytes in terms of number (versus percentage) was quantified to permit reporting “per biopsy” as was done by IHC methods. By flow, the sigmoid colonic mucosa yielded a mean of 174,939± 13,229 CD3^+^ lymphocytes per biopsy (7,288 ± 551 cells per mm^3^). Of these, 63% were CD4^+^ T lymphocytes (4,549 ± 381) and 37% CD8^+^ T lymphocytes (2,708 ± 245 cells per mm^3^) (Fig 3d). These yields (and SEM) are similar to previously reported yields by flow cytometry. Compared to the densities of total CD3^+^ lymphocytes per biopsy in sigmoid colon biopsies using IHC, flow cytometry yields were 79.3% lower. When compared to IHC-counted CD4^+^ and CD8^+^ T lymphocyte subsets per biopsy, flow cytometry yields were approximately 77.6% and 81.9% lower respectively, (Fig 4).

## Discussion

It is believed that the gastrointestinal tract and gut-associated lymphoid tissue (GALT) contain the majority of total body lymphocytes [16]. The small intestine (~4–5 meters long; internal diameter ~3 cm) has a mucosa more densely populated with immune cells than the colon including more organized lymphoid structures (Peyer’s patches (PP)) while the colon (~5 feet in length; internal diameter 5cm) has similarly diffuse lymphocytes (both intraepithelial lymphocytes (IEL) and lamina propria lymphocytes (LPL)) but lacks PP, instead having more loosely organized lymphoid aggregates (LA). Different segments of the small (duodenum, jejunum, ileum) and large (ascending, transverse, descending, sigmoid, rectum) bowel show regional heterogeneity in the organization and density of these cells; these are factors that must be taken into consideration in study design and hypothesis testing.

Using automated approaches we quantified T cells *in situ*, to determine their numbers and distribution in biopsies from healthy distal colon and rectal mucosal compartments. Mucosal compartments have been under increasing focus in the field of HIV prevention, and in inflammatory bowel diseases such as ulcerative colitis and Crohn’s disease.

Despite the common assumption that most human T lymphocytes reside in the gut, there are surprisingly few data directly addressing this issue. We sought to define absolute numbers and characterize locations of T lymphocytes in distal human colorectal mucosa by direct visualization of mucosal tissue *in situ* using frozen sections and automated quantification techniques. With this direct data, we derived estimates of the numbers of resident CD3^+^, CD4^+^ and CD8^+^ T lymphocytes in the sigmoid as well as rectal mucosal compartments. Using these data as our baseline gold standard, we demonstrate significant losses in yields of quantified T lymphocyte subsets using flow cytometry.

Overall, the direct counting identifies similar numbers of CD3+ lymphocytes in sigmoid and rectal mucosal with similar ratios of subsets: ~21K CD4^+^ and ~15K CD8^+^ T lymphocytes per mm^3^, respectively, in sigmoid mucosa and ~22K CD4^+^ and ~17K CD8^+^ T lymphocytes per mm^3^, respectively, in rectal mucosa, There was no significant difference between subsets within or between sigmoid/rectal mucosa.

Our first intention was to determine absolute numbers present in biopsies from healthy individuals to provide normative ranges for comparison in disease conditions. Having the absolute numbers enables a more informed extrapolation of the total number of T lymphocytes in these biopsies to the regional compartments. Second, by comparing direct and optimized automated counting algorithms, confidence can now be derived when utilizing this much more efficient approach than the very time-consuming and technician-dependent direct quantifications.

Thirdly, these IHC quantifications ‘per biopsy’ permitted comparisons to yields via flow cytometry ‘per biopsy’ (using TruCount beads). Importantly, for CD3^+^ as well as CD4^+^ and CD8^+^ T lymphocyte subsets, it appears ~79% of these lymphocytes phenotypes are lost after biopsy acquisition, MMC isolation, staining, fixation and flow cytometric analyses. No efforts were made here to identify which steps might be accounting for the losses and one slight limitation was the inability to obtain biopsies from a subject to freeze and manually count to use as a direct comparison to flow cytometry isolation which would have controlled for person to person variation. However, we did address this in a previous study were we describe results from four isolations performed on the same donor at the same time to document inter subject variation in MMC phenotypes and we did not find any significant differences [10]. While flow cytometry is useful for multiparametric analysis of cells in suspension [2], there is a trade-off not only in the obvious loss of information about the relative anatomic relationships of different cells, but also potentially a significant selection bias in extrapolating flow-derived results back to resident-cell populations in tissues. As well, it has been demonstrated that conventional collagenase and isolation methods disrupt and substantially reduce the expression of macrophage markers and several T lymphocyte markers such as CD3, CD4, and CD8 [17–20]. We have previously shown that PBMCs put through the same protocol we use here to isolate MMCs does not demonstrate significantly different CD3/CD4/CD8 expression compared to the PBMC’s stained prior to the 2–3 hour, collagenase and staining process [21].

Whether most of the total body human CD4^+^ T lymphocytes reside in the gut has been called into question. This concept is mostly based on extrapolations from animal studies [7, 22–23], and there are surprisingly few human data. In a review and editorial concerning the total body distribution of CD4^+^ T lymphocytes, Ganusov and De Boer suggested that ~11% of CD4^+^ T lymphocytes reside in the gut, including ~6.5% in the gut lamina propria [6]. However, this report combined only estimates from prior reviews by Westermann and Pabst [5] on B lymphocyte and CD4^+^ and CD8^+^ T lymphocyte ratios in the small intestine and from Trepel [4] on numbers of total lymphocytes in various tissue compartments (extrapolated from rats) [6]. More recently, Di Mascio *et al* proposed higher total body estimates CD4^+^ T lymphocytes but similar gut percentages, based on using radiotracer-tagged anti-CD4 monoclonal antibodies in macaques to directly quantify.

Here we report, using direct/automated counting, that the healthy human sigmoid and rectal mucosa contain about 1.38 ± 0.09 x 10^9^ and 2.62 ± 0.04 x 10^8^ CD4^+^ T lymphocytes, or about 1.6 x 10^9^ total. Assuming these sections account for ~5% of the digestive tract [24] and accounting for the greater density of lymphocytes in small bowel, these numbers fall within range of more recently published estimates [7,14].

Immunohistochemical analysis of tissues, while currently limited in the number of simultaneous markers that can be assessed, remains the gold standard in terms of both anatomic/physical representation but also absolute numbers. Recently, the analysis of IHC images has become routinely semi-automated with the aid of image processing software [25–26]. This approach, much less operator-dependent, has been used with some success to study heterogeneous populations of human tumors [27–29]. Although semi-automated analysis of IHC requires a great degree of standardization and optimization initially, it can achieve a quantitative and reliable output of protein expression on cells *in situ*, as in quantifying HER2/ER receptors on breast cancer cells in tumors [28–30]. Each specific application requires standardization of parameters such as staining threshold, the size and roundness of the nuclei and cells, and potentially other parameters such as cell elongation for each tissue and detection antibody [26].

Having accurate, clinically-relevant numbers of target cells in vulnerable but also immunologically-active compartments are essential for the evolving field of targeted therapies (including vaccines, treatment, cure efforts) as well as more conventional compartment drug delivery focused on certain cell types. Both the absolute numbers quantified and awareness of the significant losses of cells assessed by flow cytometry are important steps. While conducting these descriptive but important comparisons would have been preferable using the same study subjects, this would have been resource-intensive (both human and financial). Given the large number of subject’s flow cytometry results available and the intensity of the detailed IHC (manual and automated) counting, we felt this was a merited, leveraged, pilot investigation for CD3^+^, CD4^+^ and CD8^+^ T lymphocyte populations.

In summary, we find that healthy human colorectal mucosa is rich in T lymphocytes with specific anatomic relationships of their CD4^+^ and CD8^+^ subsets, and provide solid estimates of the densities and numbers of these cells. Additionally, we demonstrate that mucosal cell isolation by current conventional techniques recovers a minority of the mucosal T lymphocytes identified by flow cytometry after a standard tissue isolation protocol. Consequently, flow cytometric analyses must be interpreted with caution and ideally complemented with IHC studies of cells *in situ*. Accurate delineation of leukocytes in human mucosal tissues will be pivotal in understanding mucosal immunity as it relates to the pathogenesis of infections and autoimmune diseases in this compartment, as well as the development of vaccines and therapeutics applicable to mucosal immunity.

## Supporting Information

S1 Fig Calculations for lymphocyte counts. Calculations based on the average diameter of a T lymphocyte to determine amount of sections that will constitute a whole T lymphocyte in rectal and colonic tissue.
(TIF)

S2 Fig CD45 Bio-distribution in normal colorectal mucosal tissue. Sigmoid colonic and rectal mucosal sections immunostained for CD45. The membrane red/brown colors denote positive staining (red/brown, AEC chromogen). Specimens were counterstained with hematoxylin. (A) Magnification 200x (B) 400x.
(TIF)
