# Supplementary figures and images for: T Lymphocyte Density and Distribution in Human Colorectal Mucosa, and Inefficiency of Current Cell Isolation Protocols
Source: PLoS One. 2015 Apr 9;10(4):e0122723. doi: 10.1371/journal.pone.0122723 (PMC4391713; doi:10.1371/journal.pone.0122723)

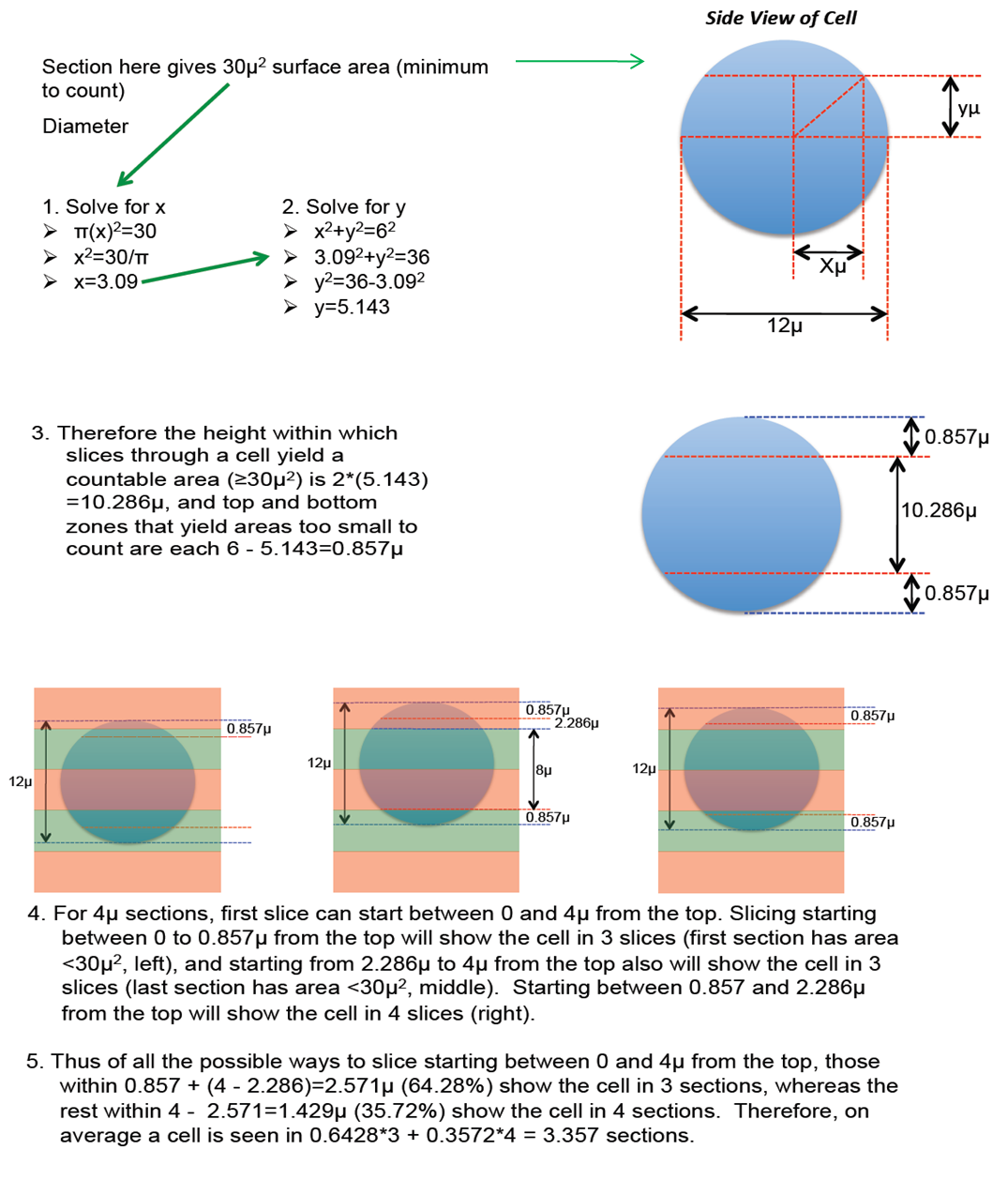

Supplement: S1 Fig — Calculations based on the average diameter of a T lymphocyte to determine amount of sections that will constitute a whole T lymphocyte in rectal and colonic tissue. (TIF) [file pone.0122723.s001.tif]

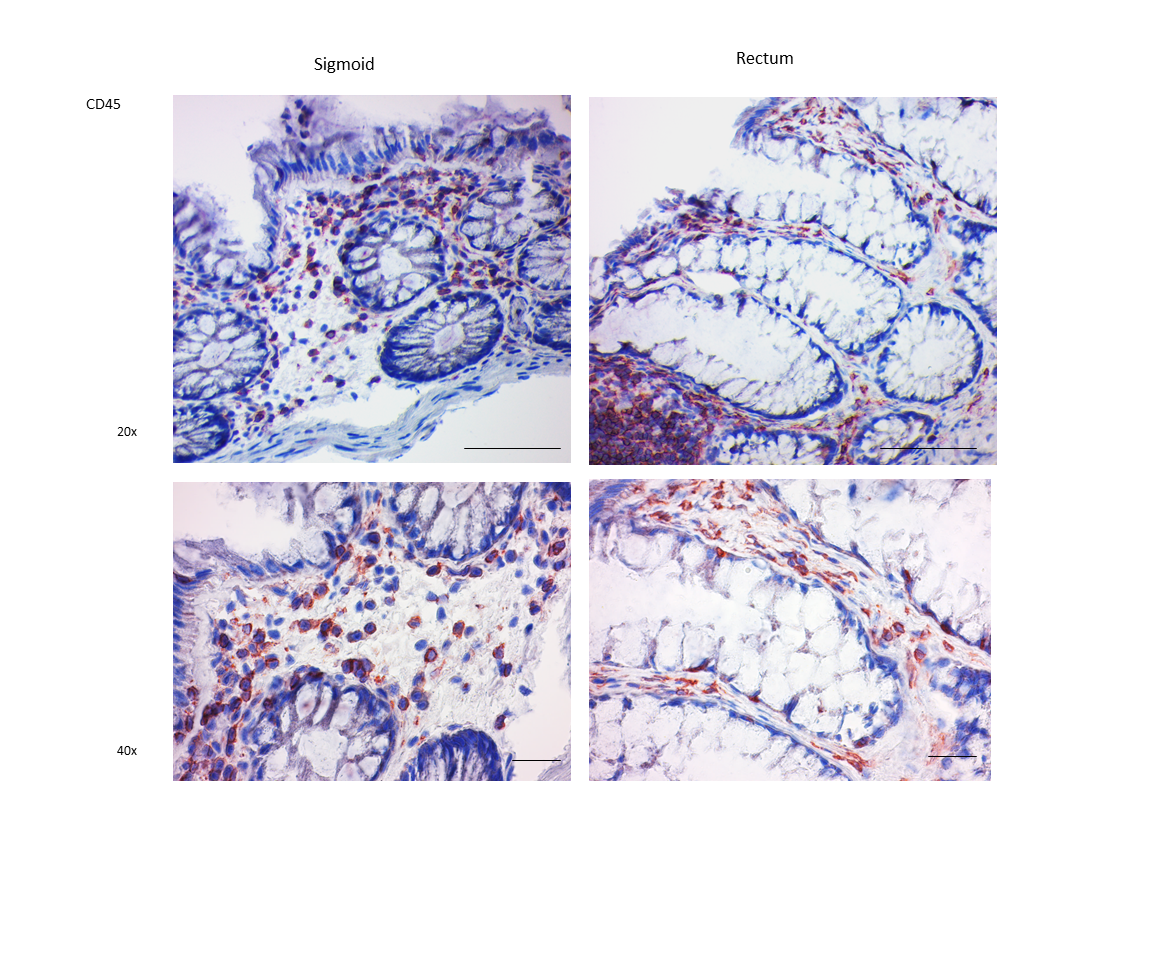

Supplement: S2 Fig — Sigmoid colonic and rectal mucosal sections immunostained for CD45. The membrane red/brown colors denote positive staining (red/brown, AEC chromogen). Specimens were counterstained with hematoxylin. (A) Magnification 200x (B) 400x. (TIF) [file pone.0122723.s002.tif]
